# Higher levels of IL-1ra, IL-6, IL-8, MCP-1, MIP-3α, MIP-3β, and fractalkine are associated with 90-day mortality in 132 non-immunomodulated hospitalized patients with COVID-19

**DOI:** 10.1371/journal.pone.0306854

**Published:** 2024-07-10

**Authors:** Liv Rabøl Andersen, Bettina Hindsberger, Simone Bastrup Israelsen, Lise Pedersen, Pal Bela Szecsi, Thomas Benfield

**Affiliations:** 1 Center of Clinical Research and Disruption of Infectious Diseases (CREDID), Department of Infectious Diseases, Copenhagen University Hospital—Amager and Hvidovre, Hvidovre, Denmark; 2 Department of Clinical Biochemistry, Holbaek Hospital, Holbaek, Denmark; 3 Department of Clinical Medicine, Faculty of Health and Medical Sciences, University of Copenhagen, Copenhagen, Denmark; Universitat de Valencia Facultat de Medicina i Odontologia, SPAIN

## Abstract

**Introduction:**

Immune dysregulation with an excessive release of cytokines has been identified as a key driver in the development of severe COVID-19. The aim of this study was to evaluate the initial cytokine profile associated with 90-day mortality and respiratory failure in a cohort of patients hospitalized with COVID 19 that did not receive immunomodulatory therapy.

**Methods:**

Levels of 45 cytokines were measured in blood samples obtained at admission from patients with confirmed COVID-19. Logistic regression analysis was utilized to determine the association between cytokine levels and outcomes. The primary outcome was death within 90 days from admission and the secondary outcome was need for mechanical ventilation.

**Results:**

A total of 132 patients were included during the spring of 2020. We found that one anti-inflammatory cytokine, one pro-inflammatory cytokine, and five chemokines were associated with the odds of 90-day mortality, specifically: interleukin-1 receptor antagonist, interleukin-6, interleukin-8, monocyte chemoattractant protein-1, macrophage inflammatory protein-3α, macrophage inflammatory protein-3β, and fractalkine. All but fractalkine were also associated with the odds of respiratory failure during admission. Monocyte chemoattractant protein-1 showed the strongest estimate of association with both outcomes.

**Conclusion:**

We showed that one anti-inflammatory cytokine, one pro-inflammatory cytokine, and five chemokines were associated with 90-day mortality in patients hospitalized with COVID-19 that did not receive immunomodulatory therapy.

## Introduction

The clinical course of COVID-19 is remarkably varied, ranging from no or mild symptoms to severe pneumonia and death [1]. Among the clinical risk factors for COVID-19 related mortality are age, male sex, cardiovascular disease, chronic pulmonary disease, diabetes, obesity, malignancies, and immunosuppression [2]. Furthermore, a dysregulated immune response has been identified as a key driver in the development of severe disease [3, 4]. The immune dysregulation is characterized by an uncontrolled excessive release of cytokines and secondary organ damage. This phenomenon, designated cytokine release syndrome or cytokine storm (CS), has been recognized since the early 90s as an overreaction of the immune system in the presence of a pathogen or inflammation [5]. Similar conditions have also been observed in other viral pneumonias, such as severe acute respiratory syndrome (SARS) and avian influenza [6, 7]. CS can cause a wide range of symptoms, including acute respiratory distress syndrome, vasodilatory shock, and disseminated vascular coagulation [5], all complications that have also been reported in cases of severe COVID-19 [8, 9]. Accordingly, suppression of the immune system by steroids, such as dexamethasone, has shown a non-specific beneficial effect in COVID-19 patients with hypoxemia [10]. More specific immunomodulatory treatments, such as the interleukin-6 (IL-6) receptor antibody, tocilizumab, and Janus kinase (JAK) inhibitor, baricitinib, have also proven beneficial in treating selected groups of patients with severe COVID-19 [11, 12].

Cytokines can be defined as soluble proteins that are secreted from a vast number of different cells and mediate communication and recruitment between different parts of the immune system [13]. Some have pro- or anti-inflammatory properties and a subgroup, chemokines, facilitate the recruitment of leukocytes to sites of inflammation or injury [13, 14]. In CS, different cell types, signaling pathways, and cytokines are involved in the pathogenesis dependent on the underlying cause [5]. Thus, several studies have investigated which cytokines are associated with adverse outcomes in SARS-CoV-2 infection. For some cytokines, such as IL-6, interleukin-8 (IL-8), interleukin-10 (IL-10), and tumor necrosis factor-α (TNF-α), which are among the most commonly studied cytokines, there is a well-established association between elevated levels and a higher risk of mortality [15–17], while other cytokines remain less investigated. In this study, we sought to evaluate the cytokine profile associated with clinical outcomes of COVID-19. We performed an analysis of 45 cytokines and their association with 90-day mortality and respiratory failure in a cohort of patients hospitalized with COVID-19 that did not receive any immunomodulatory treatment.

## Methods

### Study design

Our study is a prospective, single-center cohort study of patients diagnosed with COVID-19 pneumonia, admitted to Copenhagen University Hospital–Amager and Hvidovre from March 16 to May 19, 2020. During this period, the Wuhan variant of SARS-CoV-2 was dominant in Denmark [18]. Details of the cohort have been previously described [19]. In short, all consecutive patients were included if they were ≥ 18 years, had a positive reverse transcriptase polymerase chain reaction for SARS-CoV-2 on an oropharyngeal swab or tracheal aspirate, and had an available blood sample obtained within four days of admission. Patients received the best supportive care at the time, including intensive care, invasive mechanical ventilation, extracorporeal membrane oxygenation, and continuous renal replacement therapy. The follow-up time was 90 days from admission. Information on age, sex, body mass index, comorbidities, clinical characteristics, vital parameters, laboratory parameters, and vital status at follow-up were manually extracted from electronic health records and managed using Research Electronic Data Capture browser-based software (REDCap, Vanderbilt, TN, USA). Serum was separated from blood by centrifugation and stored at -80° C.

### Ethical considerations

The study was approved by the Regional Data Protection Center (P-2020-262), the Danish Patient Safety Authority (record no. 31-1521-309), and by the Regional Committee on Health Research Ethics (H-20040649). The requirement of individual informed consent was waived by the committee.

### Cytokine measurements

Serum samples were retrospectively analyzed for 45 cytokines by multiplex immunoassays performed with minor modifications according to the manufacturer instructions using magnetic fluorescently labeled microsphere beads with suspension array system 45-plex Fixed Panel (LKTM014) (R&D Systems, Abingdon, UK) and analyzed on a BioPlex 200 instrument (Bio-Rad, Hercules, California). Analytes measured were: Monocyte chemoattractant protein-1 (CCL2/JE/MCP-1); Macrophage inflammatory protein-1α (CCL3/MIP-1α); Macrophage inflammatory protein-1β (CCL4/MIP-1β); Regulated upon activation, normal T-cell expressed, and presumably secreted (CCL5/RANTES); Eotaxin (CCL11); Macrophage inflammatory protein-3α (CCL20/MIP-3α); Macrophage inflammatory protein-3β (CCL19/MIP-3β); TNF ligand superfamily member 5 (CD40 Ligand/TNFSF5); Fractalkine (CX3CL1); Growth regulated oncogene-α (CXCL1/GRO-α/KC/CINC-1); Growth regulated oncogene-β (CXCL2/GRO-β/MIP-2/CINC-3); Interferon-inducible protein 10 (CXCL10/IP-10/CRG-2); Epidermal growth factor (EGF); Fibroblast growth factor (FGF basic/FGF2/bFGF); FMS-like tyrosine kinase-3 ligand (Flt-3 ligand/FLT3L); Granulocyte colony stimulating factor (G-CSF); Granulocyte-macrophage colony stimulating factor (GM-CSF); Granule enzyme B (Granzyme B); Interferon-α2 (IFN-α2/IFNA2); Interferon-β (IFN-β); Interferon-γ (IFN- γ); Interleukin-1α (IL-1α /IL-1F1); Interleukin-1β (IL-1β/IL-1F2); Interleukin-1 receptor antagonist (IL-1ra/IL-1F3); Interleukin-2 (IL-2); Interleukin-3 (IL-3); Interleukin-4 (IL-4); Interleukin-5 (IL-5); Interleukin-6 (IL-6); Interleukin-7 (IL-7); Interleukin-8 (IL-8/CXCL8); Interleukin-10 (IL-10); Interleukin-12p70 (IL-12p70); Interleukin-13 (IL-13); Interleukin-15 (IL-15); Interleukin-17 (IL-17/IL-17A); Interleukin-17E (IL-17E/IL-25); Interleukin-33 (IL-33); Programmed death-1 ligand 1 (PD-L1/B7-H1); Platelet derived growth factor-AA (PDGF-AA); Platelet derived growth factor-AB/BB (PDGF-AB/BB); Transforming growth factor-α (TGF-α); Tumor necrosis factor-α (TNF-α); TNF-related apoptosis inducing ligand (TRAIL/TNFSF10); Vascular endothelial growth factor (VEGF).

For each of the analytes, the lower and upper limit of quantification were defined as the lowest/highest measurable standard +/- three times the standard deviation. Values below or above these limits were assigned a value of 10% lower or higher than the limit of quantification.

### Outcomes

The primary outcome was death within 90 days from blood sampling. The secondary outcome was respiratory failure defined as receiving mechanical ventilation during admission.

### Statistics

Categorical variables are presented as counts with percentages and continuous variables as medians with interquartile range (IQR). Comparisons between patients with and without available samples and between survivors and non-survivors were performed with χ^2^-test, Fisher’s exact test or Mann Whitney *U* test, as appropriate. Correlations were estimated by Spearman’s rank correlation coefficient. Logistic regression analyses were performed to assess whether circulating cytokine levels were associated with outcomes. Results are presented as crude and adjusted odds ratios (ORs) with 95% confidence intervals (CIs). Covariates included in the adjusted analyses were age group, sex, and the presence of ≥ 1 comorbidity (hypertension, acute myocardial infarction, heart failure, diabetes, chronic obstructive pulmonary disease, and asthma). Adjustment variables were chosen as they were previously reported as major risk factors for COVID-19 related mortality [2, 20]. Also, they had to be available for > 90% of subjects. As cytokine levels were not normally distributed, they were log2-transformed prior to analysis, and thus the estimate reflects the OR associated with a doubling of cytokine levels. Age was divided into groups; ≤ 60 years, 61–80 years, ≥ 81 years based on reports that the association between age and mortality is nonlinear [2]. For analyses with respiratory failure as the outcome, subjects with a do-not-intubate order were omitted. To select which cytokines to include in the logistic regression analyses, we applied Bonferroni correction to adjust for multiple comparisons. As 45 cytokines were tested, p-value was set at 0.0011. For all other analyses, a p-value < 0.05 was considered statistically significant. Data management and analyses were carried out using R software program version 4.0.3 [21].

## Results

### Study population

A total of 324 patients were admitted with COVID-19 during the study period. Of these, 132 patients had an available blood sample drawn within four days of admission and were eligible for inclusion in our study. The median time from admission to sampling was two days (IQR 2–3). Included subjects had a median age of 72 years (IQR 59 years– 81 years), more were male, and many were overweight. The majority had at least one comorbidity, of which hypertension and diabetes were the most commonly reported. At admission, most subjects presented with pulmonary consolidation on chest X-ray and half required supplemental oxygen. Elevated levels of plasma C-reactive protein, ferritin, and lactate dehydrogenase were also common findings (Table 1). Individuals with available cytokine samples were comparable to individuals without available cytokine samples on many parameters, including age, body mass index, and comorbidities (S1 Table). However, the two groups were different regarding sex and general condition at admission, as included subjects were more ill with a higher respiratory rate, lower saturation, higher temperature, and elevated markers of inflammation and organ damage (S1 Table).

**Table 1:** Characteristics, vital parameters, and laboratory values at admission of non-survivors and survivors.

	Non-survivors (n = 41)	Survivors (n = 91)	Total (n = 132)	p-value
Age, median [IQR]	79 [71, 84]	65 [53, 76]	72 [59, 81]	**< 0.001**
Age group, n (%)				
≤ 60 years	2 (4.9)	38 (41.8)	40 (30.3)	
61–80 years	20 (48.8)	37 (40.7)	57 (43.2)	
≥ 81 years	19 (46.3)	16 (17.6)	35 (26.5)	**< 0.001**
Sex, n (%)				
Female	17 (41.5)	38 (41.8)	55 (41.7)	
Male	24 (58.5)	53 (58.2)	77 (58.3)	1.00
Body mass index, median [IQR]^a^	28.2 [24.2, 31.2]	28.2 [24.4, 31.9]	28.2 [24.3, 31.7]	0.88
Comorbidity, n (%)				
Any	32 (78.0)	63 (69.2)	95 (72.0)	0.40
Hypertension	27 (65.9)	36 (39.6)	63 (47.7)	**< 0.01**
Acute myocardial infarction/heart failure	3 (7.3)	8 (8.8)	11 (8.3)	1.00
Diabetes	15 (36.6)	25 (27.5)	40 (30.3)	0.40
Chronic obstructive pulmonary disease	5 (12.2)	6 (6.6)	11 (8.3)	0.46
Asthma	6 (14.6)	10 (11.0)	16 (12.1)	0.76
Pulmonary infiltrate on chest X-ray, n (%)	37 (90.2)	73 (80.2)	110 (83.3)	0.24
Supplemental oxygen, n (%)	25 (61.0)	42 (46.2)	67 (50.8)	0.17
Vital parameters, median [IQR]				
Respiratory rate (/min)^b^	23 [20, 30]	20 [18, 26]	22 [18, 28]	0.13
Saturation (%)	95 [91, 96]	95 [94, 98]	95 [93, 97]	0.09
Heart rate^c^	88 [79, 98]	87 [78, 98]	87 [78, 98]	0.81
Systolic blood pressure (mmHg)	135 [117, 145]	129 [116, 141]	130 [116, 142]	0.51
Temperature (C°)^d^	38.2 [37.5, 38.8]	38.1 [36.9, 38.8]	38.1 [37.0, 38.8]	0.29
Laboratory values, median [IQR]				
Lymphocytes (10^9^/L)^e^	0.8 [0.6, 1.2]	1.0 [0.8, 1.3]	1.0 [0.7, 1.2]	**0.03**
Platelets (10^9^/L)^e^	193 [154, 224]	201 [173, 268]	199 [168, 251]	0.14
C-reactive protein (mg/L)^e^	104 [51, 168]	96 [51, 149]	98 [51, 153]	0.31
Ferritin (μg/L)^b^	689 [347, 1683]	454 [216, 1086]	620 [263, 1243]	**0.04**
Lactate dehydrogenase (U/L)^f^	394 [269, 507]	318 [237, 415]	333 [250, 446]	0.05
Alanine aminotransferase (U/L)^e^	33 [27, 53]	32 [21, 55]	32 [23, 54]	0.42
Urea (mmol/L)^g^	8.9 [7.5, 11.9]	5.7 [4.0, 7.9]	6.7 [4.2, 9.7]	**< 0.001**
Creatinine (μmol/L)^e^	102 [90, 133]	84 [69, 103]	91 [75, 111]	**< 0.001**

Any comorbidity: ≥ 1 of the following: hypertension, acute myocardial infarction and/or heart failure, diabetes, chronic obstructive pulmonary disease, and asthma

^a^ Body mass index was missing for 20 subjects.

^b^ Respiratory rate and ferritin were missing for 1 subject.

^c^ Heart rate was missing for 2 subjects.

^d^ Temperature was missing for 3 subjects.

^e^ Lymphocytes, platelets, C-reactive protein, alanine aminotransferase, and creatinine were missing for 19 subjects.

^f^ Lactate dehydrogenase was missing for 26 subjects.

^g^ Urea was missing for 20 subjects.

At the 90-day follow up, 41 patients (31%) had died (Table 1). Compared to survivors, non-survivors were older and more had hypertension. At admission, non-survivors presented with higher levels of ferritin, urea, and creatinine as well as a lower lymphocyte count. Respiratory failure resulting in mechanical ventilation occurred in 21 patients during admission (16%).

### Cytokine levels and their association with 90-day mortality

**Fig 1:**
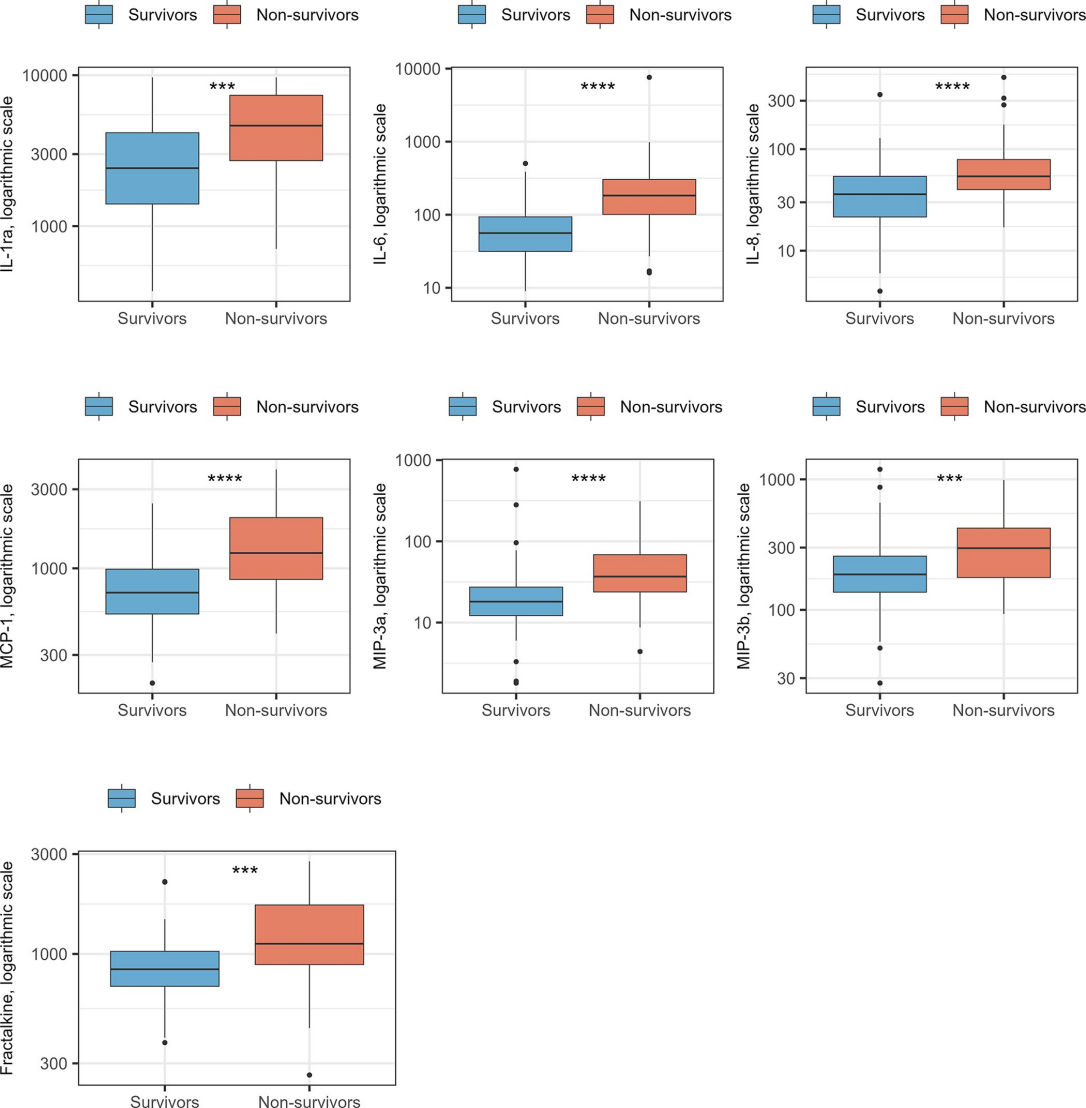
Boxplots on the distribution of cytokine levels among survivors and non-survivors. The cytokine levels are presented on a log-10 scale (y-axis). Statistics are performed by Mann Whitney *U* test. ****: p < 0.0001 and ***: p < 0.001. Abbreviations: IL-1ra: interleukin-1 receptor antagonist; IL-6: interleukin-6; IL-8: interleukin-8; MCP-1: monocyte chemoattractant protein-1; MIP-3a: macrophage inflammatory protein-3α; MIP-3b: macrophage inflammatory protein-3β.

Of the 45 analyzed cytokines, 38 were measurable in serum, while IL-3, IL-4, IL-5, IL-17A, IL-17E, IFN-β, and FGF-basic were outside the limit of quantification. After Bonferroni correction, seven cytokines were found to be significantly different between survivors and non-survivors and were thus subjected to further analysis. The selected seven cytokines were pro-inflammatory IL-6, anti-inflammatory IL-1ra, and chemokines MCP-1, IL-8, MIP-3α, MIP-3β, and fractalkine (Fig 1 and S2 Table).

**Fig 2:**
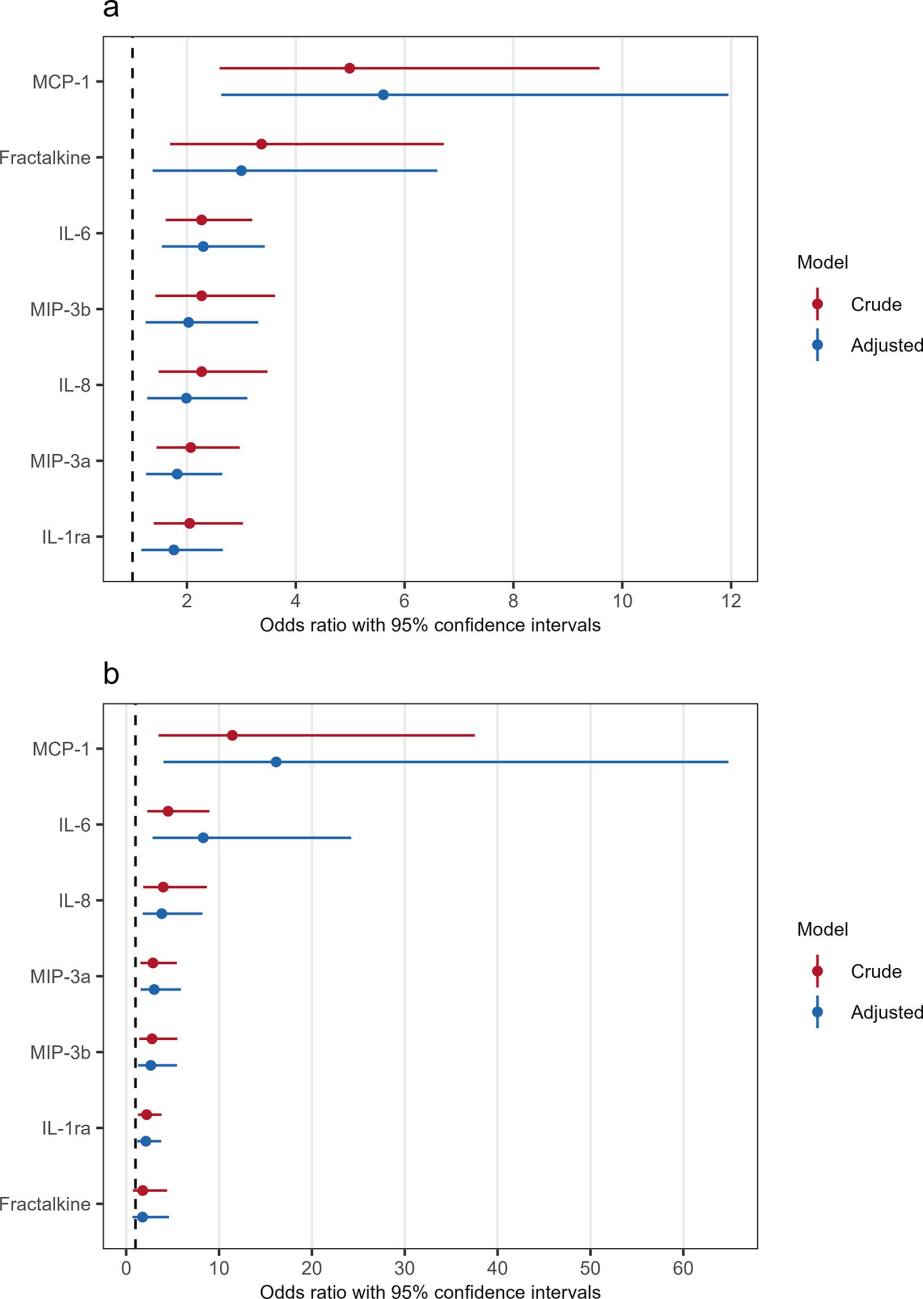
Forest plot on the associations between cytokine levels and 90-day mortality and respiratory failure. The association between cytokine levels at admission and 90-day mortality (a) and respiratory failure (b) in patients hospitalized with COVID-19. Adjusted models are adjusted for age groups, sex, and the presence of ≥ 1 comorbidity. Each estimated odds ratio corresponds to the odds ratio associated with a doubling of cytokine levels. Abbreviations: IL-1ra: interleukin-1 receptor antagonist; IL-6: interleukin-6; IL-8: interleukin-8; MCP-1: monocyte chemoattractant protein-1; MIP-3a: macrophage inflammatory protein-3α; MIP-3b: macrophage inflammatory protein-3β.

When applying logistic regression, all seven cytokines were also associated with 90-day mortality in crude analyses. MCP-1 was associated with the highest OR of 90-day mortality (OR 4.99, CI 2.60–9.58), corresponding to a five-fold increase in the odds of 90-day mortality per doubling of circulating MCP-1 levels. Analysis of fractalkine provided the second largest estimate (OR 3.37, CI 1.69–6.72). In a model adjusted for age group, sex, and comorbidity, the association between increasing cytokine levels and 90-day mortality persisted (Fig 2). Again, levels of MCP-1 were associated with the highest odds of 90-day mortality (OR 5.61, CI 2.63–11.95), followed by fractalkine (OR 3.00, CI 1.37–6.60) and IL-6 (OR 2.30, CI 1.54–3.43). In addition, survival curve analyses stratified by quartiles of cytokine levels were applied (S1A-S1G Fig). The figures illustrate that the risk of 90-day mortality increases with higher levels of cytokines and that especially subjects with cytokine levels above the upper quartile have an increased risk of 90-day mortality with a survival probability of 50% or lower.

### Cytokine levels and their association with respiratory failure

IL-1ra, IL-6, IL-8, MCP-1, MIP-3α, MIP-3β, and fractalkine were investigated regarding respiratory failure leading to mechanical ventilation during admission. For these analyses, subjects with a do-not-intubate order were omitted. For all cytokines, except for the chemokine fractalkine, higher circulating levels were associated with respiratory failure in crude logistic regression analyses. The highest estimates were found for MCP-1 (OR 11.43, CI 3.48–37.55), IL-6 (OR 4.51, CI 2.27–8.96), and IL-8 (OR 3.99, CI 1.83–8.69). Similar results were found when applying a model adjusted for age group, sex, and comorbidity. The association between respiratory failure and higher levels of IL-1ra, IL-6, IL-8, MCP-1, MIP-3α, and MIP-3β persisted, and again MCP-1 was the cytokine with the highest estimate (OR 16.15, CI 4.02–64.86), followed by IL-6 (OR 8.30, CI 2.84–24.23), and IL-8 (OR 3.83, CI 1.78–8.21).

### Correlation analyses

When analyzing all pairwise correlations between IL-1ra, IL-6, IL-8, MCP-1, MIP-3α, MIP-3β, and fractalkine, the cytokines mostly exhibited low to moderate positive correlations (S2 Fig). The strongest correlations were found between the pairs IL-1ra & IL-6 (0.58), IL-6 & MCP-1 (0.56), MIP-3α & MIP-3β (0.56), and IL-8 & MCP-1 (0.55), all p < 0.001, while there was no correlation between IL-6 & fractalkine. Generally, only no or low correlations were found among the cytokines and other laboratory parameters, the strongest being among MIP-3α & urea (0.46), MCP-1 & lactate dehydrogenase (0.44), IL-1ra & urea (0.43), and fractalkine & urea (0.43), all p < 0.001 (S2 Fig).

## Discussion

Cytokines and chemokines are an important part of our immune defense. These short-lived proteins mediate communication, amplification, activation, and recruitment within the immune system. However, as important as they are for fighting pathogenic organisms, an overzealous response can be harmful for the host. During the SARS-CoV-2 pandemic, a large proportion of patients with severe COVID-19 has showed signs of cytokine storm. With the recent advance in producing targeted antibody therapy against several cytokines, a better understanding of the dysregulated cytokine production during COVID-19 is warranted. Hence, in our study, we measured the serum levels of 45 cytokines in a cohort of hospitalized patients with COVID-19 and examined their association with 90-day mortality and respiratory failure. We showed that one anti-inflammatory cytokine, one pro-inflammatory cytokine, and five chemokines were associated with the odds of 90-day mortality, specifically IL-1ra, IL-6, IL-8, MCP-1, MIP-3α, MIP-3β, and fractalkine. Of these, all except fractalkine were also associated with the odds of respiratory failure. MCP-1 showed the strongest estimate of association with both outcomes, with a five-fold increase in the odds of 90-day mortality and a 16-fold increase in the odds of respiratory failure in adjusted analyses. Generally, there existed low to moderate positive correlations between pairs of cytokines, while there was only no or low correlations between cytokines and other laboratory parameters.

In our study, IL-6 was consistently associated with outcomes in both crude and adjusted analyses. IL-6 is perhaps the most investigated cytokine in the context of COVID-19, and in line with our results, a large number of studies and several meta-analyses have concluded that increases in IL-6 levels are associated with COVID-19 severity and mortality [16, 17, 22]. IL-6 has pro-inflammatory properties and functions as an important mediator of the acute phase response. Elevated levels of IL-6 have been described in many cytokine storms disorders, where IL-6 is thought to play a key role in the immunopathology, and therapeutic agents that targets the IL-6 pathway have been found beneficial in some of these conditions [5]. The same goes for COVID-19, where tocilizumab, an IL-6 antagonist, and baricitinib, a JAK inhibitor, have been found to lower mortality in selected groups of severely ill patients [11, 12].

The anti-inflammatory cytokine IL-1ra was also found to be associated with both respiratory failure and mortality in our study, confirming results from other reports [23, 24]. IL-1ra is probably elevated to down-regulate the hyperinflammation, specifically IL-1 subtypes [5]–although in our study, we did not observe significantly higher levels of IL-1α or IL-1β. However, higher levels of IL-1β have been associated with admission to intensive care units in other studies [24, 25]. Considering this, treatment with anakinra, recombinant human IL-1ra, has been investigated for a potential beneficial effect in patients with COVID-19. Some studies show a beneficial effect in selected groups of patients, while others describe no difference in outcomes between anakinra and usual care [26, 27].

The five chemokines IL-8, MCP-1, MIP-3α, MIP-3β, and fractalkine were significantly associated with COVID-19 mortality in our study, and all but fractalkine were also associated with respiratory failure. For both IL-8 and MCP-1, higher levels have been associated with COVID-19 mortality and respiratory failure in a number of studies [16, 17, 25, 28, 29]. The remaining chemokines, MIP-3α, MIP-3β, and fractalkine, are generally less investigated with regard to COVID-19 outcomes and results are less consistent. A small study by Hue et al. found that MIP-3α and MIP-3β were not associated with 28-day mortality in crude and adjusted analyses, while fractalkine was only associated with 28-day mortality in crude analyses, contrasting our results [30]. However, other studies found that a fatal outcome of COVID-19 was associated with higher MIP-3α levels in plasma and in bronchoalveolar lavage fluid, respectively [31, 32]. Regarding MIP-3β, a study by Tveita et al. concluded that high levels were associated with respiratory failure, admission to intensive care units, and 60-day mortality, when utilizing survival curve analysis, which is in line with our results [33]. Also, a multi-omics analysis identified MIP-3α and MIP-3β as two of the main discriminatory features in identifying a cluster of patients with a higher risk of 28-day mortality [34]. In the case of fractalkine, another two studies conclude that circulating levels were higher among non-survivors than survivors and in patients with severe disease compared to mild disease [35, 36]. The discrepancy between the studies might be due to the small sampling size in the study by Hue et al., limiting statistical power.

Some cytokines, such as TNF-α and IL-10, have been associated with COVID-19 severity and mortality in other studies [16, 37, 38], but was not found to be significantly different between survivors and non-survivors after Bonferroni correction in our study. Infliximab, a TNF-α inhibitor, was shown to lower 28-day mortality in a selected group of patients with COVID-19, suggesting a role of TNF-α in the pathogenesis of more severe COVID-19 [39].

Overall, our study showed that levels at admission of several cytokines with both pro-inflammatory, anti-inflammatory, and chemotactic properties are associated with respiratory failure and mortality in patients with COVID-19. Thus, the hyperinflammation associated with SARS-CoV-2 is rather complex, as described for other cytokine storm syndromes [5]. Several different therapies, including steroids, IL-6-inhibitors, JAK-inhibitors, IL-1 receptor antagonists, and TNF-α inhibitors, have been reported to be beneficial in the treatment of selected groups with COVID-19. This indicates that many molecules can function as drug targets in COVID-19 associated cytokine storm if they are part of central inflammatory pathways. Reducing the general levels of circulating cytokines through hemoadsorption, remains more controversial, as some studies report a decrease in mortality, while others have found no improvement or even negative effects on survival [40, 41].

Our study was strengthened by the consecutive enrollment of patients, a complete follow-up, and a large study size. Furthermore, the study was carried out during the spring of 2020 where no immunomodulatory treatment regimens for COVID-19 had been established. Thus, the levels of circulating cytokines were unlikely to have been influenced by the administration of dexamethasone or other immunomodulatory therapy. However, there are also some limitations to consider. Given the observational nature of the study, we have only been able to assess association and not causality. Cytokines were measured as multiplex with only a single dilution, and therefore many cytokines were outside of the measurable range. These cytokines may have an undiscovered association with outcomes of COVID-19. It should also be noted that our study was primarily conducted on patients admitted to the Department of Infectious Diseases, which tended to care for more severely ill patients than other wards. Still, patients in our study were similar to patients in the overall cohort on many parameters.

In conclusion, we found that levels at admission of one pro-inflammatory cytokine, one anti-inflammatory cytokine, and five chemokines were associated with 90-day mortality in hospitalized patients with COVID-19. Besides supporting existing knowledge on especially IL-1ra, IL-6, IL-8, and MCP-1 in relation to COVID-19, our study has provided additional insight into lesser investigated chemokines associated with adverse outcomes in COVID-19 patients.

## Supporting information

S1 Table Characteristics, vital parameters, and laboratory values at admission of patients with and without available cytokine samples. Any comorbidity: at least one of the following: hypertension, acute myocardial infarction and/or heart failure, diabetes, chronic obstructive pulmonary disease, and asthma.
(DOCX)

S2 Table Median cytokine levels of non-survivors and survivors and crude odds ratios for 90-day mortality. Table sorted by odds ratios.
(DOCX)

S1 Fig Mortality and levels of cytokines at admission. Survival probability stratified by quartiles of cytokine levels at admission in patients hospitalized with COVID-19. Statistics are performed by log rank test for equal mortality in the four groups. (a) interleukin-1 receptor antagonist; (b) interleukin-6; (c) interleukin-8; (d) monocyte chemoattractant protein-1; (e) macrophage inflammatory protein-3α; (f) macrophage inflammatory protein-3β; (g) fractalkine.
(PDF)

S2 Fig Correlation matrix of cytokines and laboratory values. Correlations were estimated by Spearman’s rank correlation coefficient. Blue dots indicate a positive correlation and red dots indicate a negative correlation. The size of the dot and the intensity of the color indicate the strength of the correlation. ALT: alanine aminotransferase; CRP: C-reactive protein; IL-1ra: interleukin-1 receptor antagonist; IL-6: interleukin-6; IL-8: interleukin-8; LDH: lactate dehydrogenase; MCP-1; monocyte chemoattractant protein-1; MIP-3a: macrophage inflammatory protein-3α; MIP-3b: macrophage inflammatory protein-3β.
(PDF)
