# Supplementary material for: Higher levels of IL-1ra, IL-6, IL-8, MCP-1, MIP-3α, MIP-3β, and fractalkine are associated with 90-day mortality in 132 non-immunomodulated hospitalized patients with COVID-19
Source: PLoS One. 2024 Jul 10;19(7):e0306854. doi: 10.1371/journal.pone.0306854 (PMC11236197; doi:10.1371/journal.pone.0306854)
Supplement: S1 Table — Any comorbidity: at least one of the following: hypertension, acute myocardial infarction and/or heart failure, diabetes, chronic obstructive pulmonary disease, and asthma. (DOCX) [file pone.0306854.s001.docx]

**S1 Table. Characteristics, vital parameters, and laboratory values at admission of patients with and without available cytokine samples**

|  | **Patients excluded**  **(n = 192)** | **Patients included**  **(n = 132)** | **Total**  **(n = 324)** | **p-value** |
| --- | --- | --- | --- | --- |
| Age, median [IQR] | 73 [58, 82] | 72 [59, 81] | 72 [58, 81] | 0.92 |
| Age group, n (%) |  |  |  |  |
| ≤ 60 years | 50 (26.0) | 40 (30.3) | 90 (27.8) |  |
| 61 – 80 years | 87 (45.3) | 57 (43.2) | 144 (44.4) |  |
| ≥ 81 years | 55 (28.6) | 35 (26.5) | 90 (27.8) | 0.70 |
| Sex, n (%) |  |  |  |  |
| Female | 104 (54.2) | 55 (41.7) | 159 (49.1) |  |
| Male | 88 (45.8) | 77 (58.3) | 165 (50.9) | **0.04** |
| Body mass index, median [IQR]^a^ | 26.1 [23.0, 31.5] | 28.2 [24.3, 31.7] | 27.2 [23.5, 31.6] | 0.08 |
| Comorbidity, n (%) |  |  |  |  |
| Any | 119 (62.0) | 95 (72.0) | 214 (66.0) | 0.08 |
| Hypertension | 80 (41.7) | 63 (47.7) | 143 (44.1) | 0.33 |
| Acute myocardial infarction/heart failure | 28 (14.6) | 11 (8.3) | 39 (12.0) | 0.13 |
| Diabetes | 46 (24.0) | 40 (30.3) | 86 (26.5) | 0.25 |
| Chronic obstructive pulmonary disease | 22 (11.5) | 11 (8.3) | 33 (10.2) | 0.47 |
| Asthma | 14 (7.3) | 16 (12.1) | 30 (9.3) | 0.20 |
| Pulmonary infiltrate on chest X-ray, n (%) | 156 (81.2) | 110 (83.3) | 266 (82.1) | 0.74 |
| Supplemental oxygen, n (%) | 52 (28.1) | 67 (50.8) | 119 (37.5) | **< 0.001** |
| Vital parameters, median [IQR] |  |  |  |  |
| Respiratory rate (/min)^b^ | 20 [18, 22] | 22 [18, 28] | 20 [18, 24] | **< 0.001** |
| Saturation (%)^b^ | 96 [95, 98] | 95 [93, 97] | 96 [94, 98] | **< 0.01** |
| Heart rate^c^ | 86 [74, 99] | 87 [78, 98] | 87 [76, 98] | 0.47 |
| Systolic blood pressure (mmHg)^d^ | 133 [119, 142] | 130 [116, 142] | 132 [118, 142] | 0.28 |
| Temperature (Cº)^e^ | 37.7 [36.9, 38.4] | 38.1 [37.0, 38.8] | 37.9 [37.0, 38.5] | **0.01** |
| Laboratory values, median [IQR] |  |  |  |  |
| Lymphocytes (10^9^/L)^f^ | 1.0 [0.8, 1.5] | 1.0 [0.7, 1.2] | 1.0 [0.7, 1.4] | 0.19 |
| Platelets (10^9^/L)^g^ | 197 [158, 244] | 199 [168, 251] | 199 [162, 248] | 0.40 |
| C-reactive protein (mg/L)^h^ | 51 [24, 101] | 98 [51, 153] | 65 [35, 127] | **< 0.001** |
| Lactate dehydrogenase (U/L)^i^ | 251 [210, 336] | 333 [250, 446] | 278 [220, 383] | **< 0.001** |
| Alanine aminotransferase (U/L)^j^ | 26 [18, 41] | 32 [23, 54] | 29 [20, 45] | **0.01** |
| Urea (mmol/L)^k^ | 6.0 [4.0, 9.9] | 6.7 [4.2, 9.7] | 6.2 [4.2, 9.8] | 0.29 |
| Creatinine (µmol/L)^l^ | 84 [65, 111] | 91 [75, 111] | 86 [68, 111] | 0.36 |

Any comorbidity: at least one of the following: hypertension, acute myocardial infarction and/or heart failure, diabetes, chronic obstructive pulmonary disease, and asthma. ^a^ Body mass index was missing for 76 subjects. ^b^ Respiratory rate and saturation were missing for 7 subjects. ^c^ Heart rate was missing for 8 subjects. ^d^ Systolic blood pressure was missing for 6 subjects. ^e^ Temperature was missing for 12 subjects. ^f^ Lymphocytes was missing for 46 subjects. ^g^ Platelets was missing for 43 subjects. ^h^ C-reactive protein was missing for 44 subjects. ^i^ Lactate dehydrogenase was missing for 66 subjects. ^j^ Alanine aminotransferase was missing for 47 subjects. ^k^ Urea was missing for 49 subjects. ^l^ Creatinine was missing for 40 subjects.
