# Supplementary material for: Higher levels of IL-1ra, IL-6, IL-8, MCP-1, MIP-3α, MIP-3β, and fractalkine are associated with 90-day mortality in 132 non-immunomodulated hospitalized patients with COVID-19
Source: PLoS One. 2024 Jul 10;19(7):e0306854. doi: 10.1371/journal.pone.0306854 (PMC11236197; doi:10.1371/journal.pone.0306854)
Supplement: S2 Table — Table sorted by odds ratios. (DOCX) [file pone.0306854.s002.docx]

**S2 Table. Median cytokine levels of non-survivors and survivors and crude odds ratios for 90-day mortality**

| **Cytokine** (pg/mL) | **Non-survivors**  **(n = 41)**  Median [IQR] | **Survivors**  **(n = 91)**  Median [IQR] | **Total**  **(n = 132)**  Median [IQR] | **p-value** | **Odds ratio**  **(95% CI)** |
| --- | --- | --- | --- | --- | --- |
| MCP-1 | 1237 [857, 2025] | 713 [531, 989] | 835 [583, 1207] | < 0.001 | 4.99 (2.60 – 9.58) |
| Fractalkine | 1119 [890, 1714] | 845 [701, 1029] | 932 [717, 1133] | < 0.001 | 3.37 (1.69 – 6.72) |
| MIP-1β | 460 [409, 540] | 405 [346, 467] | 424 [353, 489] | < 0.01 | 2.62 (1.15 – 6.01) |
| IL-6 | 183 [101, 304] | 56 [32, 94] | 78 [39, 170] | < 0.001 | 2.27 (1.61 – 3.20) |
| IL-8 | 54 [40, 79] | 36 [22, 54] | 42 [26, 60] | < 0.001 | 2.27 (1.48 – 3.48) |
| MIP-3β | 297 [177, 423] | 187 [137, 258] | 201 [149, 343] | < 0.001 | 2.27 (1.42 – 3.62) |
| Flt-3 Ligand | 137 [117, 157] | 122 [99, 153] | 127 [99, 153] | 0.06 | 2.14 (0.94 – 4.89) |
| VEGF | 596 [384, 734] | 383 [252, 598] | 417 [295, 660] | < 0.01 | 2.13 (1.31 – 3.45) |
| GM-CSF | 135 [108, 155] | 113 [72, 134] | 120 [83, 139] | < 0.01 | 2.11 (1.17 – 3.80) |
| MIP-3α | 37 [24, 69] | 18 [12, 27] | 22 [14, 36] | < 0.001 | 2.07 (1.44 – 2.97) |
| IL-15 | 5 [3, 8] | 4 [3, 5] | 4 [3, 6] | < 0.01 | 2.06 (1.16 – 3.67) |
| IL-1ra | 4634 [2717, 7357] | 2422 [1402, 4157] | 2942 [1669, 5146] | < 0.001 | 2.05 (1.39 – 3.03) |
| IL-10 | 356 [241, 550] | 280 [147, 361] | 290 [197, 391] | < 0.01 | 2.00 (1.28 – 3.12) |
| PD-L1 | 208 [171, 299] | 172 [121, 218] | 185 [134, 236] | < 0.01 | 1.99 (1.18 – 3.38) |
| IL-1β | 10 [8, 13] | 9 [7, 12] | 10 [7, 12] | 0.12 | 1.79 (0.98 – 3.27) |
| IL-7 | 14 [12, 18] | 12 [8, 16] | 13 [9, 17] | 0.04 | 1.66 (1.00 – 2.75) |
| TNF-α | 14 [13, 20] | 13 [13, 19] | 13 [13, 19] | 0.15 | 1.61 (0.97 – 2.67) |
| IP-10 | 1760 [758, 2108] | 935 [434, 1527] | 1118 [481, 1771] | 0.01 | 1.50 (1.09 – 2.05) |
| TGF-α | 33 [23, 42] | 27 [21, 37] | 27 [21, 39] | 0.07 | 1.42 (0.83 – 2.43) |
| G-CSF | 63 [49, 75] | 60 [48, 77] | 62 [49, 77] | 0.69 | 1.36 (0.81 – 2.27) |
| Eotaxin | 238 [189, 319] | 218 [165, 298] | 225 [170, 303] | 0.23 | 1.30 (0.81 – 2.08) |
| MIP-1α | 10 [5, 18] | 5 [5, 10] | 5 [5, 12] | 0.03 | 1.25 (0.92 – 1.71) |
| GRO-α | 244 [185, 359] | 219 [156, 336] | 231 [173, 342] | 0.20 | 1.23 (0.81 – 1.87) |
| IL-2 | 4 [3, 6] | 4 [3, 5] | 4 [3, 5] | 0.19 | 1.20 (0.83 – 1.75) |
| IL-12p70 | 22 [22, 22] | 22 [22, 22] | 22 [22, 22] | 0.50 | 1.14 (0.67 – 1.92) |
| IFN-α2 | 4 [4, 13] | 4 [4, 12] | 4 [4, 12] | 0.28 | 1.12 (0.85 – 1.47) |
| IL-13 | 32 [32, 32] | 32 [32, 36] | 32 [32, 36] | 0.69 | 1.08 (0.55 – 2.12) |
| Granzyme B | 16 [5, 29] | 15 [7, 22] | 15 [6, 23] | 0.47 | 1.06 (0.77 – 1.45) |
| IL-33 | 17 [13, 23] | 17 [13, 22] | 17 [13, 22] | 0.97 | 0.96 (0.52 – 1.76) |
| GRO-β | 2056 [1505, 2733] | 1919 [1278, 2811] | 1958 [1339, 2757] | 0.81 | 0.93 (0.61 – 1.40) |
| TRAIL. | 26 [23, 52] | 33 [23, 51] | 32 [23, 51] | 0.24 | 0.90 (0.58 – 1.40) |
| CD40 Ligand | 4078 [3158, 5790] | 4957 [3198, 7056] | 4940 [3151, 6485] | 0.21 | 0.85 (0.55 – 1.32) |
| RANTES | 31269 [17026, 46400] | 32057 [22283, 54548] | 31368 [21286, 52600] | 0.33 | 0.79 (0.53 – 1.17) |
| IL-1α | 16 [11, 20] | 16 [12, 20] | 16 [12, 20] | 0.80 | 0.79 (0.36 – 1.73) |
| EGF | 186 [141, 221] | 227 [153, 321] | 211 [146, 302] | 0.01 | 0.71 (0.47 – 1.09) |
| IFN-γ | 7 [7, 7] | 7 [7, 7] | 7 [7, 7] | 0.41 | 0.68 (0.27 – 1.69) |
| PDGF-AA | 5170 [5170, 5170] | 5170 [5170, 5170] | 5170 [5170, 5170] | 0.73 | 0.57 (0.16 – 2.02) |
| PDGF-AB/BB | 2430 [1916, 3998] | 3489 [2178, 5060] | 3073 [2080, 4737] | < 0.01 | 0.56 (0.35 – 0.90) |

Table sorted by odds ratios. CI: confidence interval. MCP-1: Monocyte chemoattractant protein-1, MIP-1a: Macrophage inflammatory protein-1-α; MIP-1b: Macrophage inflammatory protein-1- β; RANTES: Regulated upon activation normal T-cell expressed, and presumably secreted; MIP-3α: Macrophage inflammatory protein-3-α; MIP-3β: Macrophage inflammatory protein-3-β; CD40 Ligand: TNF ligand superfamily member 5; GRO-a: Growth regulated oncogene-α; GRO-b: Growth regulated oncogene-β; IP-10: Interferon-inducible protein 10; EGF: Epidermal growth factor; Flt-3 Ligand: Fms-like tyrosine kinase-3 ligand; G-CSF: Granulocyte colony stimulating factor; GM-CSF: Granulocyte-macrophage colony stimulating factor; Granzyme B: Granule enzyme B; IFN-a2: Interferon-α2; IFN-g: Interferon-γ; IL-1a: Interleukin-1α; IL-1b: Interleukin-1β; IL-1ra: Interleukin-1 receptor antagonist; IL-2: Interleukin-2; IL-6: Interleukin-6; IL-7: Interleukin-7; IL-8: Interleukin-8; IL-10: Interleukin-10; IL-12 p70: Interleukin-12p70; IL-13: Interleukin-13; IL-15: Interleukin-15; IL-33: Interleukin-33; PD-L1: Programmed death-1 ligand 1; PDGF-AA: Platelet derived growth factor-AA; PDGF-AB/BB: Platelet derived growth factor-AB/BB; TGF- a: Transforming growth factor-α; TNF-a: Tumor necrosis factor-α; TRAIL: TNF-related apoptosis inducing ligand; VEGF: Vascular endothelial growth factor.
