# Supplementary figures and images for: Higher levels of IL-1ra, IL-6, IL-8, MCP-1, MIP-3α, MIP-3β, and fractalkine are associated with 90-day mortality in 132 non-immunomodulated hospitalized patients with COVID-19
Source: PLoS One. 2024 Jul 10;19(7):e0306854. doi: 10.1371/journal.pone.0306854 (PMC11236197; doi:10.1371/journal.pone.0306854)

Fig. S1a (IL-1ra)

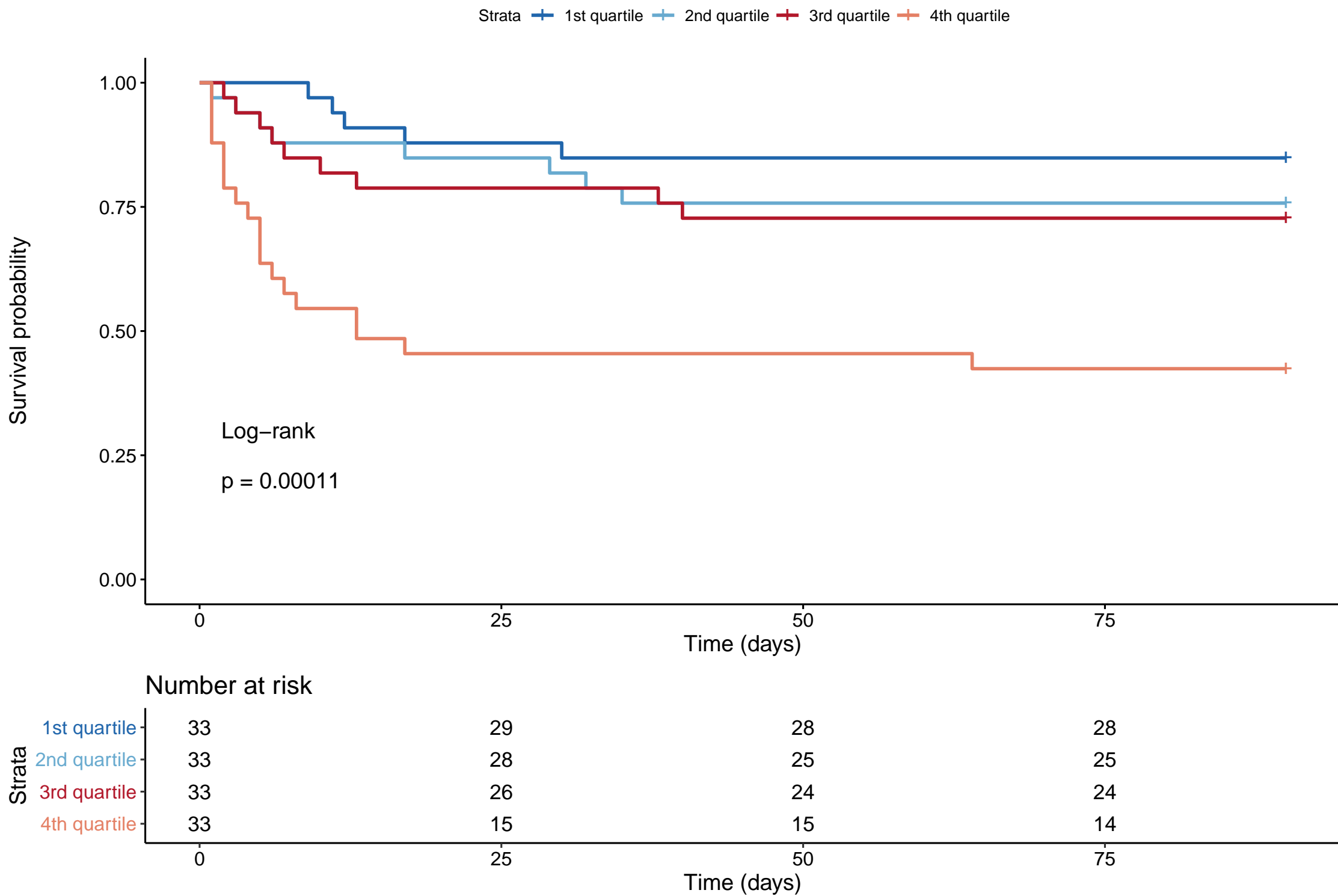

Fig. S1b (IL-6)

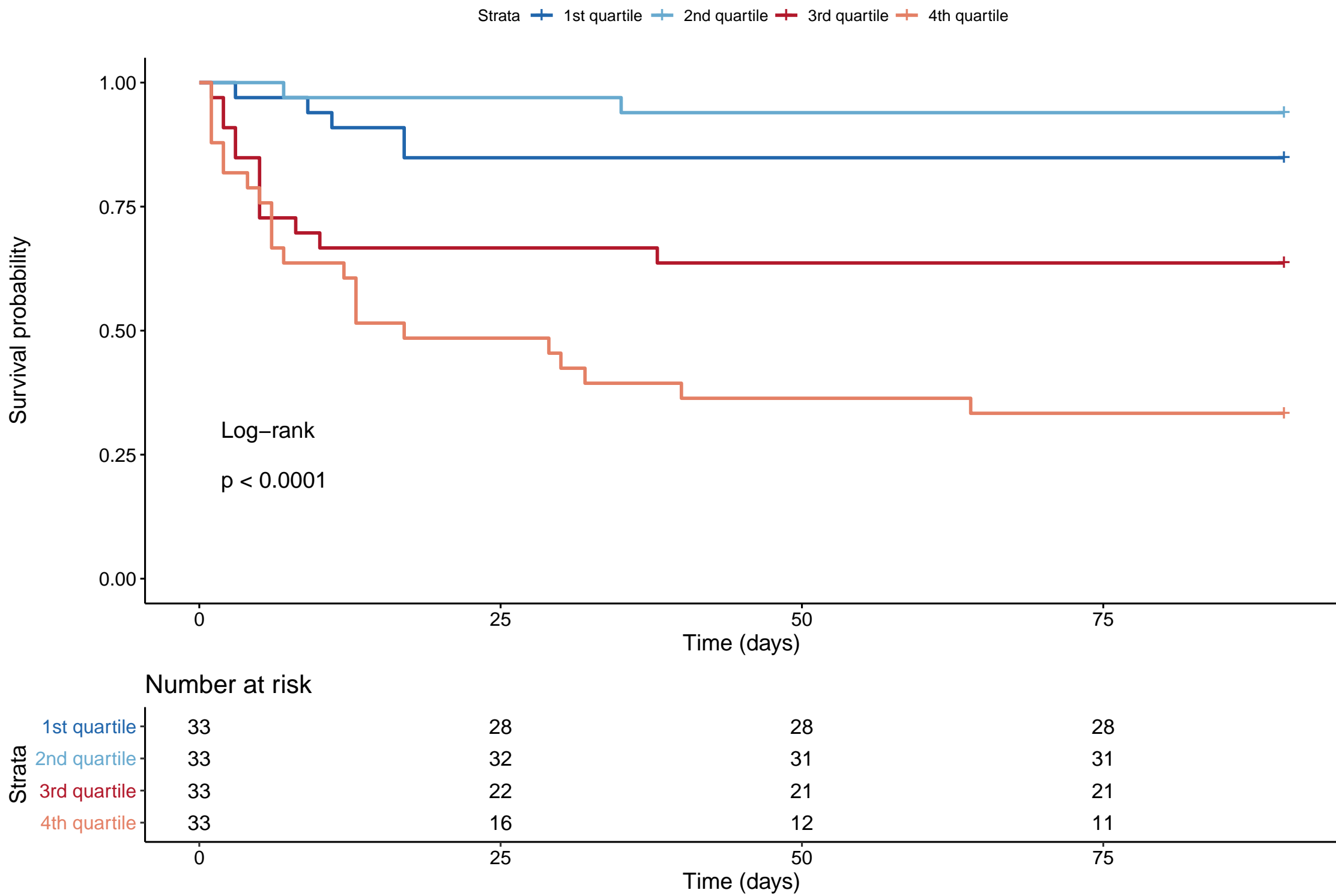

Fig. S1c (IL-8)

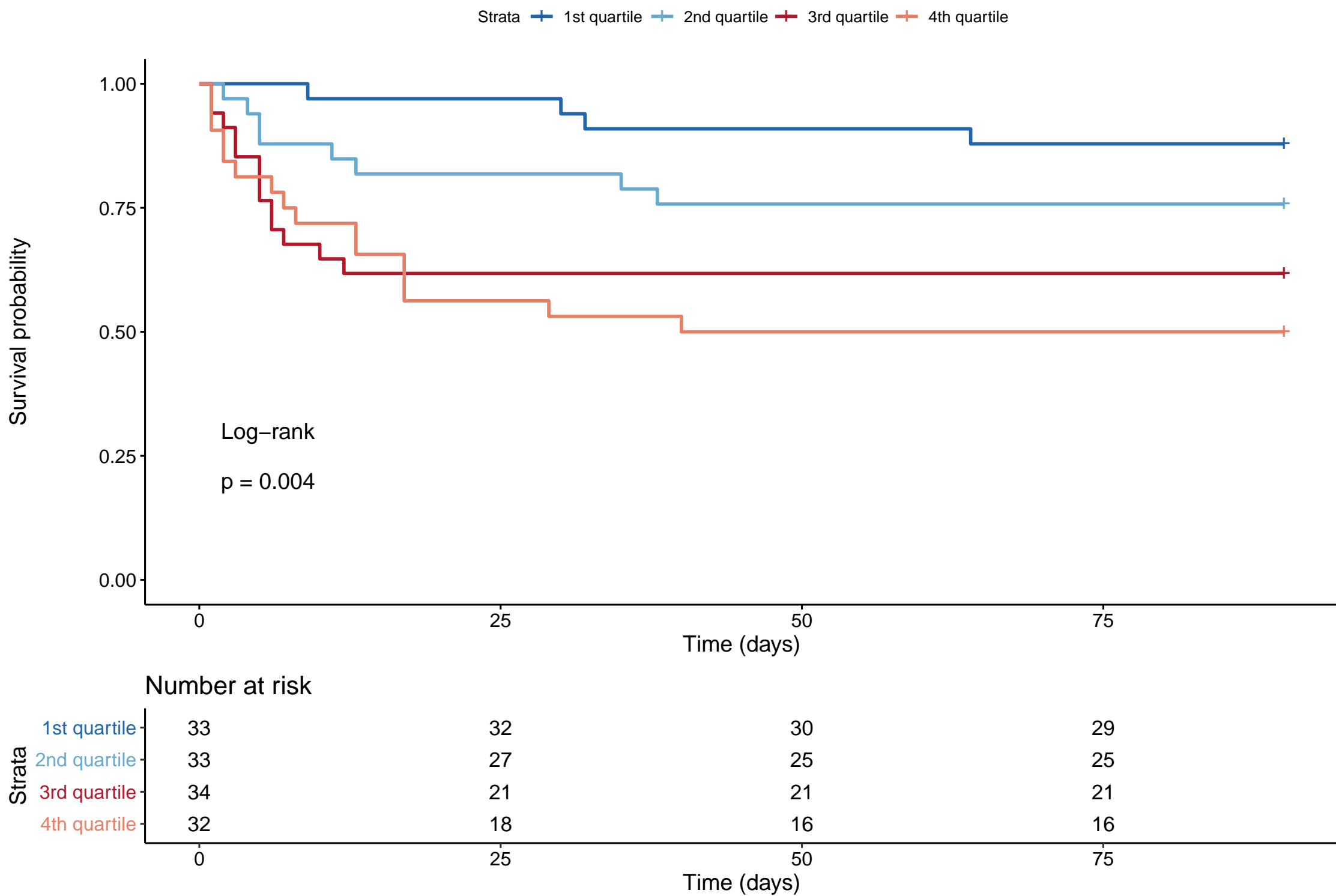

Fig. S1d (MCP-1)

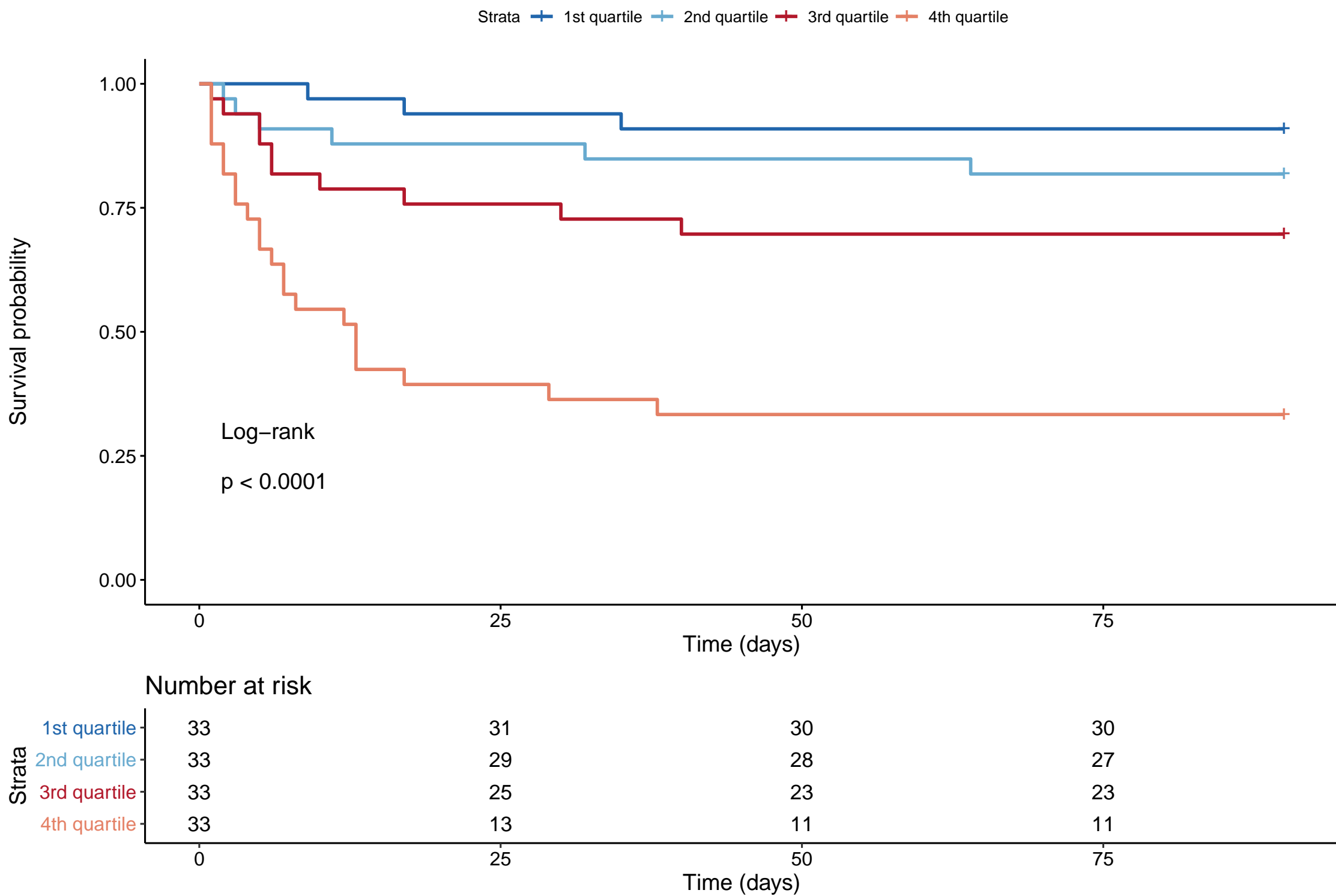

Fig. S1e (MIP-3a)

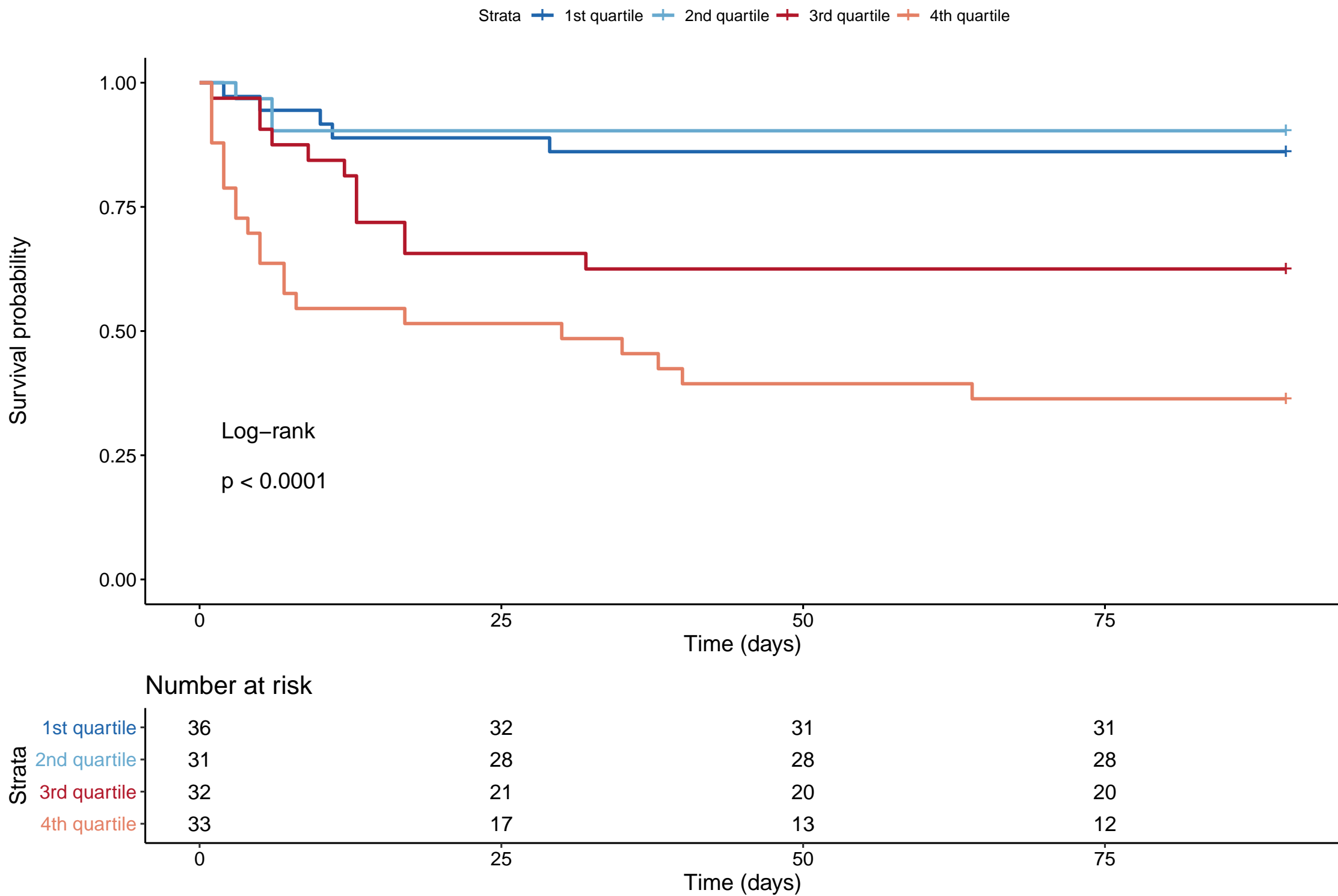

Fig. S1f (MIP-3b)

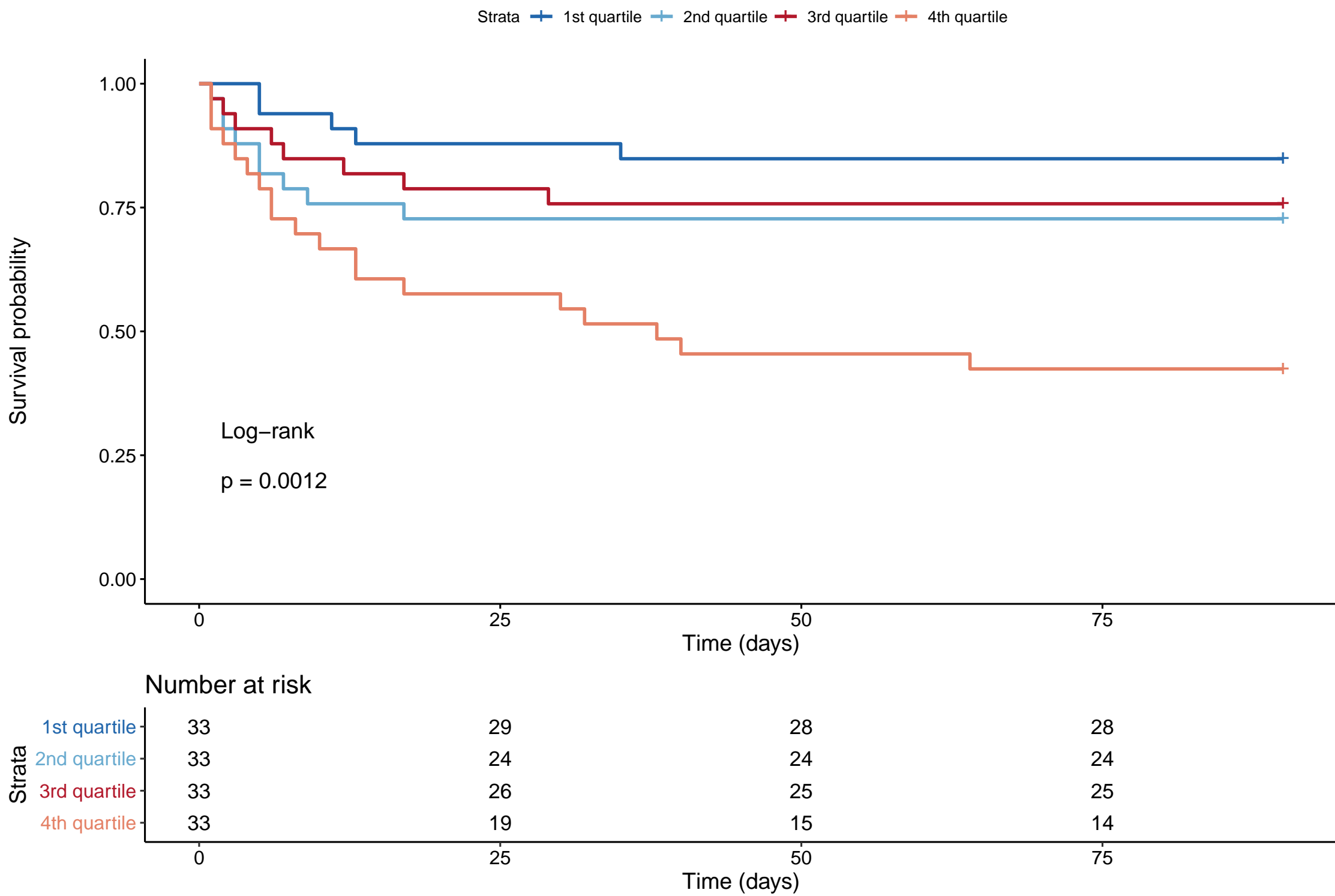

Fig. S1g (Fractalkine)

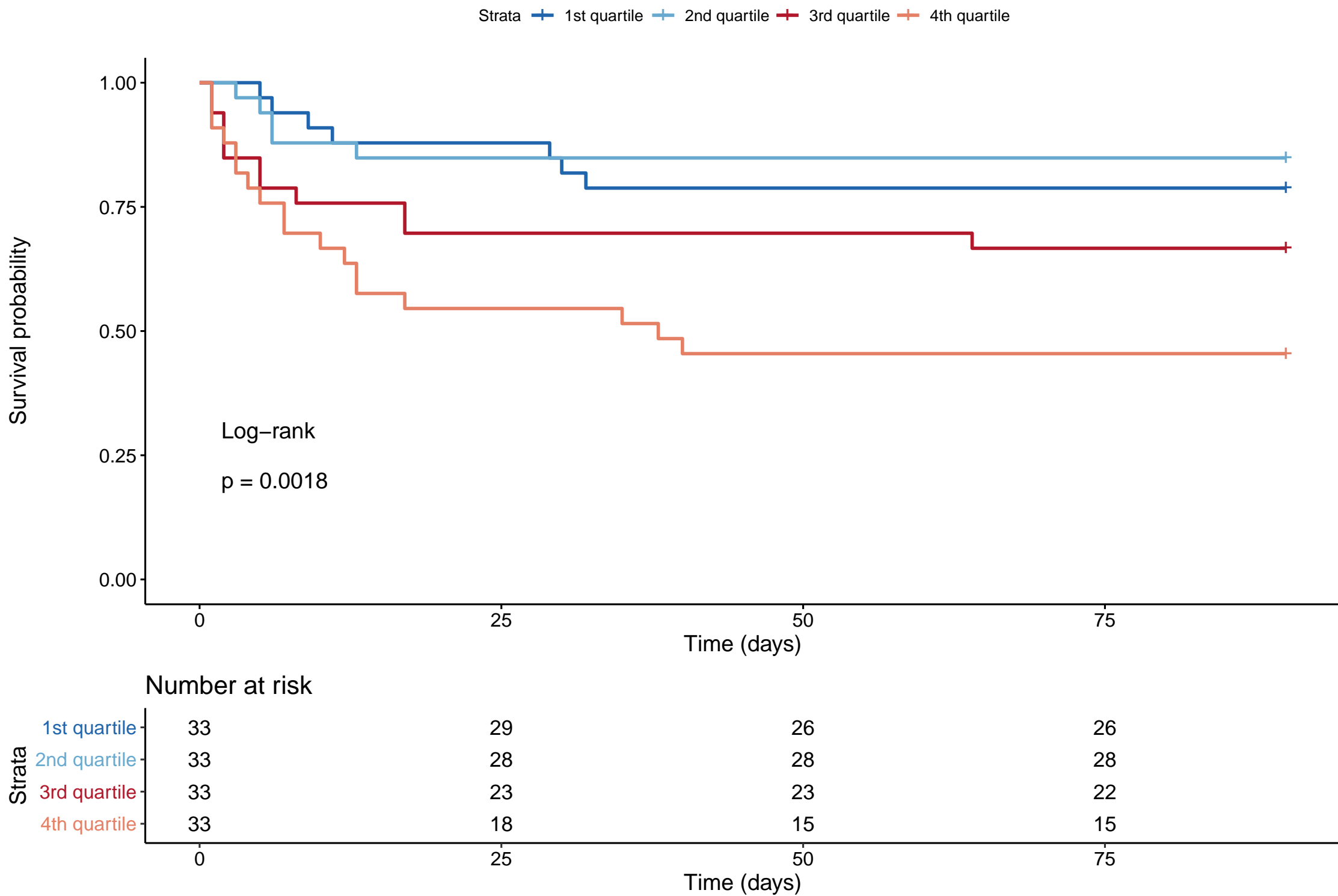

Supplement: S1 Fig — Survival probability stratified by quartiles of cytokine levels at admission in patients hospitalized with COVID-19. Statistics are performed by log rank test for equal mortality in the four groups. (a) interleukin-1 receptor antagonist; (b) interleukin-6; (c) interleukin-8; (d) monocyte chemoattractant protein-1; (e) macrophage inflammatory protein-3α; (f) macrophage inflammatory protein-3β; (g) fractalkine. (PDF) [file pone.0306854.s003.pdf]

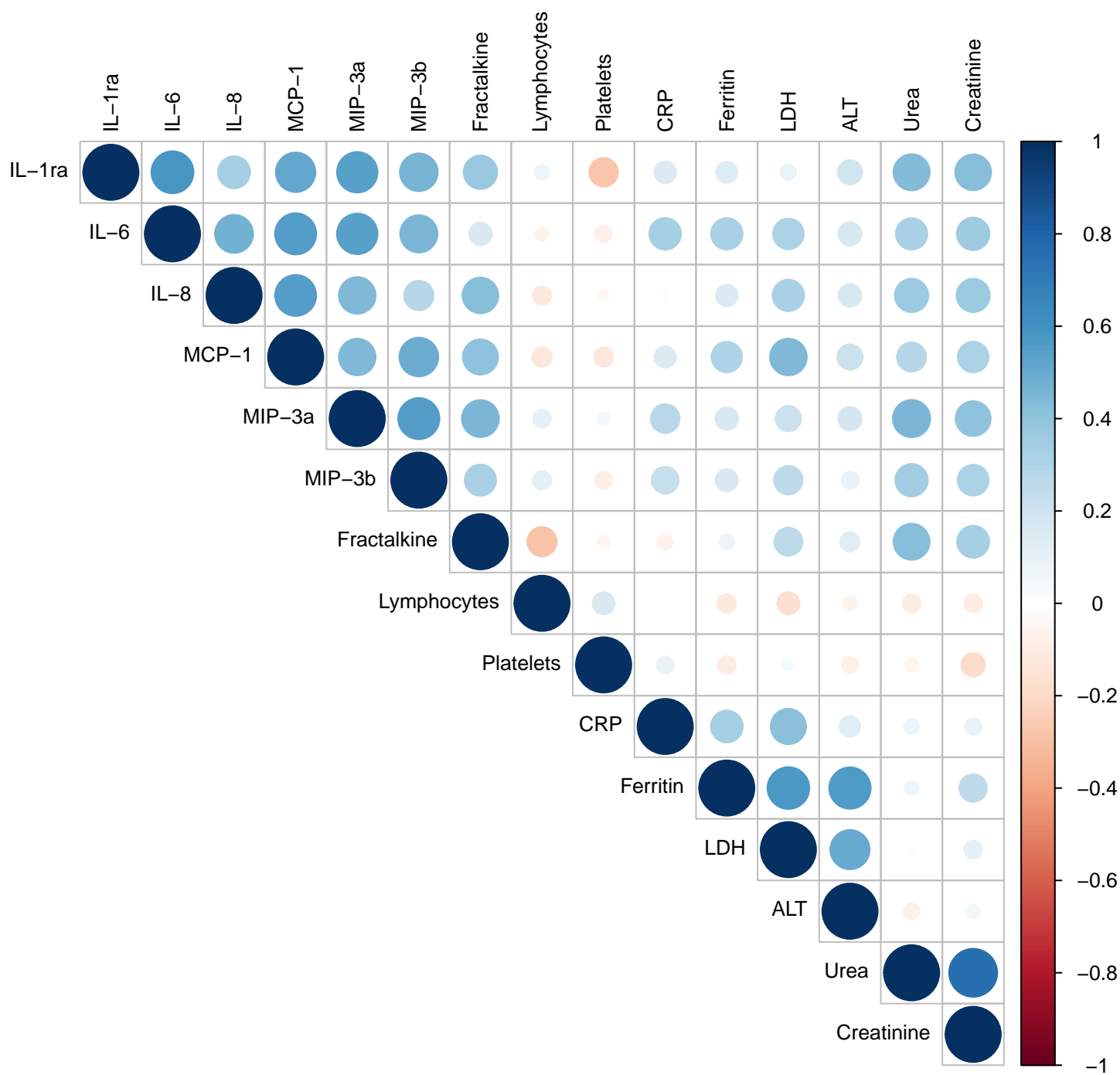

Supplement: S2 Fig — Correlations were estimated by Spearman’s rank correlation coefficient. Blue dots indicate a positive correlation and red dots indicate a negative correlation. The size of the dot and the intensity of the color indicate the strength of the correlation. ALT: alanine aminotransferase; CRP: C-reactive protein; IL-1ra: interleukin-1 receptor antagonist; IL-6: interleukin-6; IL-8: interleukin-8; LDH: lactate dehydrogenase; MCP-1; monocyte chemoattractant protein-1; MIP-3a: macrophage inflammatory protein-3α; MIP-3b: macrophage inflammatory protein-3β. (PDF) [file pone.0306854.s004.pdf]
